# Comparative cost-effectiveness analyses of cardiovascular magnetic resonance and coronary angiography combined with fractional flow reserve for the diagnosis of coronary artery disease

**DOI:** 10.1186/1532-429X-16-13

**Published:** 2014-01-25

**Authors:** Karine Moschetti, David Favre, Christophe Pinget, Guenter Pilz, Steffen E Petersen, Anja Wagner, Jean-Blaise Wasserfallen, Juerg Schwitter

**Affiliations:** 1 Institute of Health Economics and Management (IEMS), University of Lausanne, Route de Chavannes 31, VIDY, 1015 Lausanne, Switzerland; 2 Technology Assessment Unit (UET), University Hospital (CHUV), Lausanne, Switzerland; 3 Klinik Agatharied, Akademisches Lehrkrankenhaus der LMU Munich, Hausham, Germany; 4 National Institute for Health Research Cardiovascular Biomedical Research Unit at Barts, Queen Mary University of London, London, UK; 5 Comprehensive Cardiology of Stamford and Greenwich, Stamford, CT 06902, USA; 6 Cardiac MR Center, University Hospital (CHUV), Lausanne, Switzerland

**Keywords:** Cost-effectiveness analysis, Coronary artery disease, Cardiovascular magnetic resonance, Coronary angiography, Fractional flow reserve, Decision making

## Abstract

**Abstracts:**

**Background**

According to recent guidelines, patients with coronary artery disease (CAD) should undergo revascularization if significant myocardial ischemia is present. Both, cardiovascular magnetic resonance (CMR) and fractional flow reserve (FFR) allow for a reliable ischemia assessment and in combination with anatomical information provided by invasive coronary angiography (CXA), such a work-up sets the basis for a decision to revascularize or not. The cost-effectiveness ratio of these two strategies is compared.

**Methods**

Strategy 1) CMR to assess ischemia followed by CXA in ischemia-positive patients (CMR + CXA), Strategy 2) CXA followed by FFR in angiographically positive stenoses (CXA + FFR). The costs, evaluated from the third party payer perspective in Switzerland, Germany, the United Kingdom (UK), and the United States (US), included public prices of the different outpatient procedures and costs induced by procedural complications and by diagnostic errors. The effectiveness criterion was the correct identification of hemodynamically significant coronary lesion(s) (= significant CAD) complemented by full anatomical information. Test performances were derived from the published literature. Cost-effectiveness ratios for both strategies were compared for hypothetical cohorts with different pretest likelihood of significant CAD.

**Results**

CMR + CXA and CXA + FFR were equally cost-effective at a pretest likelihood of CAD of 62% in Switzerland, 65% in Germany, 83% in the UK, and 82% in the US with costs of CHF 5′794, € 1′517, £ 2′680, and $ 2′179 per patient correctly diagnosed. Below these thresholds, CMR + CXA showed lower costs per patient correctly diagnosed than CXA + FFR.

**Conclusions**

The CMR + CXA strategy is more cost-effective than CXA + FFR below a CAD prevalence of 62%, 65%, 83%, and 82% for the Swiss, the German, the UK, and the US health care systems, respectively. These findings may help to optimize resource utilization in the diagnosis of CAD.

## Background

In many countries, cardiovascular diseases are the leading cause of morbidity and also of loss of quality of life. In particular, coronary artery disease (CAD) constitutes a major public health problem. In Europe the total costs of CAD and stroke were estimated at 49 billion Euros in the year 2008 [1] and for the United States these costs were estimated as high as 156 billion dollars [2]. It is well established that patients with no evidence of myocardial ischemia have low cardiac events rates, even when invasive coronary angiography (CXA) demonstrates lesions of intermediate severity [3,4]. In addition, patients without ischemia can be treated safely with medical therapy [5,6] thereby reducing the total costs of patient management [7]. On the other hand, randomized trials e.g. in diabetic patients demonstrated a survival benefit of patients with ischemia being treated by revascularization versus medical treatment alone [8]. Accordingly, recent guidelines recommend to revascularize patients if a relevant burden of myocardial ischemia is present (i.e. proximal vessel(s)) with a positive fractional flow reserve (FFR) and/or >10% of myocardium ischemic [9] or ≥2 ischemic segments in cardiovascular magnetic resonance (CMR) perfusion examinations [10].

Nevertheless, neither vessel anatomy nor presence or absence of ischemia is the factor that will exclusively decide on revascularizations. Symptoms, co-morbidities and other factors have to be taken into account before a treatment decision is made. Thus, the current analysis was undertaken to assess the cost-effectiveness to acquire information (significant ischemia and full anatomical information) relevant for decision making, but did not include the costs for all information needed to manage patients with CAD. Also, we do not know whether a large ischemia burden is directly related to adverse effects, whether it represents a marker of higher risk for occlusion of a severe stenosis that causes ischemia, or whether more severe ischaemia is simply a marker of more extensive atherosclerosis and more vulnerable plaques that go along with a worse outcome. Large trials such as ISCHEMIA and others [11] will hopefully improve our knowledge in a near future on how to treat ischemic patients. Despite this current lack of a detailed understanding of the underlying mechanisms that link ischemia to outcome, current guidelines recommend an ischemia-based approach for decision making in patients with CAD. Therefore, the aim of the current study was to assess the cost-effectiveness of two diagnostic strategies that are ischemia-based and provide both full anatomical and functional evaluation of CAD, which are the basis for a revascularization procedure.

A variety of new imaging techniques allow for such a combined anatomical and functional assessment of CAD, and as a result, the selection of the optimum test becomes more and more challenging. The choice of cardiovascular imaging techniques should consider both, their clinical benefits for the patients as well as the costs and cost-effectiveness compared to others. Invasive coronary angiography (CXA) remains the reference for the morphological assessment of CAD and it is often used in daily practice as a first line test, e.g. in patients with a positive treadmill test. While this strategy is not recommended by current guidelines, the advent of the FFR measurement to assess the hemodynamic significance of coronary artery stenoses [12] may even increase the attractivity to use invasive CXA as a first line test, as it can be easily combined with FFR in case of intermediate stenoses. Also, the FFR results were highly predictive for patient outcome [13,14] and the combination of CXA with FFR was more cost-effective than a CXA-only approach for the treatment of CAD [15]. Accordingly, recent guidelines recommend using FFR to correctly identify lesions that should undergo percutaneous coronary interventions (PCI) [9]. However, the invasive nature and radiation exposure of CXA and FFR limit their usefulness in a screening process [16]. Considering the fact that CXA is still extensively used in many industrialized countries as an early step in the work-up of suspected CAD [17,18], and further considering that FFR is recommended in recent guidelines, the combination of CXA + FFR was one diagnostic strategy to be assessed in the current study with respect to its cost-effectiveness.

As an alternative to the FFR measurement, perfusion CMR has emerged as a robust non-invasive technique for the evaluation of myocardial ischemia [19-22]. Furthermore, recent studies demonstrated the excellent prognosis of patients with known or suspected CAD, when perfusion-CMR was normal [23-25]. Accordingly, in the present study, the cost-effectiveness of a combination of CMR + CXA was compared with that of a CXA + FFR strategy.

In the current economic context, the health care systems have to be economically sustainable while preserving high quality medical standards. Consequently, in the following study we estimated the costs of the two different strategies relative to their effectiveness to 1) correctly diagnose the presence of relevant ischemia (= significant CAD) and 2) to yield full anatomical information of the coronary vasculature in case of ischemia. In particular, the cost-effectiveness of the two strategies was compared when applied to patient populations with varying CAD pre-test probabilities. Strategy 1 consists of a CMR examination to assess ischemia followed by CXA in ischemia-positive patients (CMR + CXA). This strategy yields complete information on myocardial ischemia and coronary anatomy. Strategy 2 consists of a CXA in all patients followed by a FFR test in patients with intermediate stenoses on CXA (CXA + FFR). Finally, the cost-effectiveness ratios of the two strategies were calculated for the health care systems in Switzerland, Germany, the United Kingdom, and the United States.

## Methods

**Figure 1:**
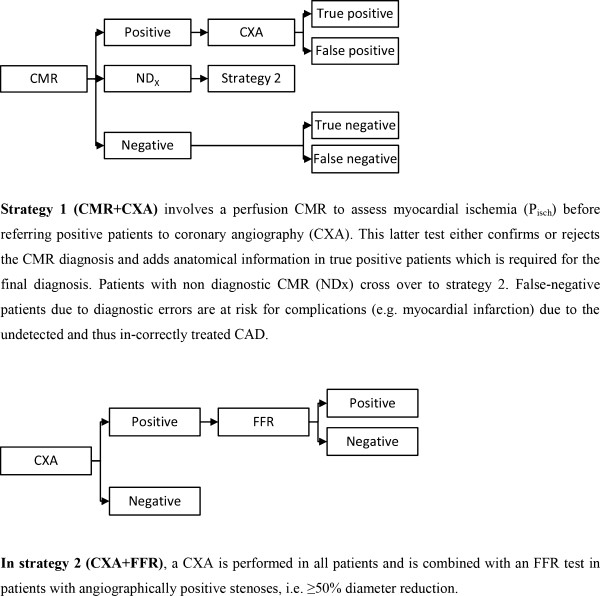
Decision tree for CAD diagnosis including strategy 1 and strategy 2 used to design the model.

Using a mathematical model, we compared the cost-effectiveness of 2 algorithms for diagnosing the presence of hemodynamically significant coronary lesion(s) (= significant CAD) for hypothetical patient cohorts characterized by different pretest likelihood of CAD (P_isch_): 1) A perfusion CMR to assess myocardial ischemia before referring positive patients to CXA and 2) A CXA combined with an FFR in patients with angiographically positive stenoses (see also Figure 1).

### Model characteristics

The model is based on Bayes’ theorem and consequently assesses cost-effectiveness ratios of strategies in hypothetical patient cohorts with different pretest likelihoods of disease [26]. The mathematical model was initially suggested by Paterson and co-workers [27] and was later on applied by others [28-30]. The simulation approach has the advantage to allow the evaluation of diagnostic algorithms for patients with different pretest likelihoods of CAD regardless of currently accepted and applied clinical strategies to detect CAD. In order to determine the pretest likelihood of CAD in patients, the same testing procedures would precede both strategies, i.e. CMR + CXA and CXA + FFR, implying the same costs for both strategies. Therefore, these “upstream” costs need not to be considered in the model. Similarly, once a treatment decision is made, based on either diagnostic strategy the same treatment costs will occur and therefore, these “downstream” costs were not considered either in the model. No ethics approval was obtained for this study as it is based on simulation model calculations and therefore no patients data from our institution were required. Calculations were performed with Microsoft Office Excel 2007 software.

### Cost-effectiveness analysis

#### Definition of effectiveness

In the present study, the criterion of effectiveness is the ability to accurately identify those patients with one or more hemodynamically significant coronary lesion(s) (=significant CAD), combined with the complete anatomical information on the coronary arteries. These patients with a relevant ischemia burden are the primary candidates for revascularizations according the most recent guidelines [10]. This ischemia burden is defined in the newest guidelines as a positive FFR of proximal coronary vessels [9,10] or by ≥2 segments with ischemia on perfusion-CMR [10]. The effectiveness criterion for strategy 1 is achieved by a positive perfusion-CMR study (≥2 segments ischemic, see also Table 1), which is complemented by a complete anatomical information provided by a CXA examination in patients positive for ischemia. For strategy 2, the effectiveness criterion is achieved by the detection of a stenosis ≥50% in CXA combined with an FFR ≤0.75 (= significant CAD). By assumption, invasive CXA and FFR were the reference tests with an assumed 100% diagnostic accuracy (Table 1). For the calculation of hemodynamically significant lesions by the CMR + CXA strategy, per-patient sensitivities (Sn_CMR_ = 0.88) and specificities (Sp_CMR_ = 0.90) were considered as determined by Rieber et al. who compared ischemia on CMR (i.e. ≥2 segment positive) versus FFR ≤0.75 as the reference for ischemia [31]. Cost-effectiveness is defined as the costs per effect which is calculated as the ratio between the total costs and the number of patients correctly diagnosed as having one or more hemodynamically significant coronary lesion(s) (true positive). Also, the costs of complications in patients with a false negative diagnosis are included in the cost-effectiveness ratio.

**Table 1:** Test performance parameters used in the effectiveness calculations

**Abbreviation**	**Description**	**Parameter value**
**Test to yield anatomical information (= detection of diameter reduction ≥50%)**		
**Sn**_**CXA**_	Sensitivity of CXA	1
**Sp**_**CXA**_	Specificity of CXA	1
**Sn**_**FFR**_	Sensitivity of FFR	1
**Sp**_**FFR**_	Specificity of FFR	1
**R**_**CXA**_	Rate of complications with invasive CXA	0.0005 [27]
**Test to yield ischemia information (=detection of myocardial ischemia)**		
**Sn**_**CMR**_	Sensitivity of CMR (≥2 segments positive vs FFR ≤ 0.75)	0.88 [31]
**Sp**_**CMR**_	Specificity of CMR (≥2 segments positive vs FFR ≤ 0.75)	0.90 [31]
**NDx**	Non diagnostic rate for CMR	0.05 [32]
**R**_**F**_	Rate of complications per 10-year follow-up period for patients with CAD and false-negative tests	0.15 [27]

In this study, the objective of the analysis was to compare cost-effectiveness ratios from the third party payer perspective and not to assess the general impact of CAD detection on the society welfare.

When performing cost-effectiveness analysis, a wide variety of factors and parameters related to the costs and the performances of the tests have to be considered. The model must be able to take into account the costs associated with false-positive results (i.e. costs of unnecessary diagnostic tests or treatments) as well as the costs associated with false negative results (i.e. costs of complications because of inappropriate management of the disease). To this end, data from the published literature on the performances of tests (sensitivities, specificities and rate of non-diagnostic examinations) and the complication rates were used (Table 1).

To appropriately model strategy 2 (CXA + FFR), the portion of patients with diameter stenoses ≥50% on CXA having ischemia in FFR must be known. In other words, the relationship between the probability of stenoses ≥50% on CXA (P_sten_) and the probability of having ischemia on FFR (P_isch_) must be known. In order to assess this relationship, we used published data from 5 recent articles (see Additional file 1: Section A1 for details).

### Definition of costs and calculations of the costs per effect

The costs of a diagnostic strategy consist of first-line test costs and subsequent costs. The first-line test costs are the fees (F_t_) for the CMR and CXA tests. Subsequent costs were costs of additional tests (i.e. in case of non-diagnostic CMR or unnecessary diagnostic tests in case of false-positive results), costs of major complications induced by the diagnostic procedure or resulting from mis-diagnosis of a patient (e.g. as false negative patients are at risk to have complications per 10-years follow-up because of inappropriate management of the disease). Due to the non-invasive nature of CMR and recent results showing that no severe complications occurred in >17,000 CMR examinations (i.e. 7 mild reactions in >7,200 stress CMR examinations) in the EuroCMR registry [33] and in large multicenter trials [19], we assumed that CMR is not associated with direct procedure-related major complications. As an anaphylactic shock is extremely rare and may occur after administration of both MR- and CXA-related contrast media, this complication was not considered in the analysis. We assumed that a potential complication associated with either a diagnostic CXA or an untreated hemodynamically significant lesion (i.e. false negatives) is a myocardial infarction (MI). Costs for this complication (hereafter C) included medical costs associated with the complications and accounted for a PCI, a hospital stay of one week, and a rehabilitation period of 4 weeks. The risk of developing malignancies induced by radiation exposure was not incorporated into the model. Future complications-related costs were discounted annually at a rate of 3% [34].

The total costs of a diagnostic algorithm were calculated as the sum of direct costs and subsequent costs multiplied by the respective number of patients. The cost-effectiveness ratios were calculated for patient cohorts with different pretest likelihoods ranging from 10% to 100%.

The cost-effectiveness ratios were calculated as follows:

$$\begin{array}{cc} \mathrm{Cost}-\mathrm{effectiveness} & \mathrm{Ratio} \end{array}=\frac{\begin{array}{cc} \mathrm{Direct} & \mathrm{cost} \end{array}+\begin{array}{cc} \mathrm{subsequent} & \mathrm{costs} \end{array}}{\mathrm{effectiveness}}$$

Calculations of the direct and subsequent costs and the detailed equations are presented in the Additional file 1: Section A2.

### Evaluation of the costs in each country

The analyses were conducted from the third party payer perspective in 4 countries. We used 2012 and 2013 costs data in Swiss Francs (CHF) for Switzerland, in Euros (€) for Germany, in Pounds (£) for the United Kingdom (UK), and in American Dollars (US $) for the United States. The Additional file 2 provides a brief description on how the costs were derived for each country.

### Sensitivity analysis

Due to the uncertainty of the data used and the numerous assumptions (parameter values) made in these calculations, a sensitivity analysis was performed to test the robustness of the model. Thus, the model was re-run with 1) changes in the costs of the tests and of the complications, 2) changes in the rates of complications associated with CXA, 3) changes in the accuracy of the CMR test, and 4) change in the threshold of FFR to detect ischemia (<0.75 vs <0.80; regarding the method used refer to Additional file 1). In order to understand the impact of each parameter in the model they were changed one by one in the repeated calculations (for details, see the figures related to the sensitivity analyses in the Results section).

## Results

### Effect of the pretest likelihood of significant CAD on effectiveness and on costs of the two strategies

**Figure 2:**
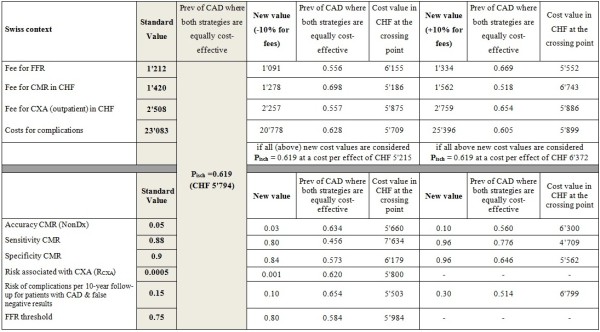
**Example for the Swiss health care system: Proportion of patients with suspected CAD correctly diagnosed (CAD Dx) by the CMR + CXA and CXA + FFR strategies in relation to pre-test likelihood of significant CAD (P**_**isch.**_**)**

Figure 2 shows the effect of the pretest likelihood of significant CAD on effectiveness. The proportion of patients with CAD for whom a correct diagnosis is made by the CMR-based strategy depends on its sensitivity, specificity, and the rate of non-diagnostic CMR examinations (Additional file 1: Section A2). As CXA and FFR are assumed to be the reference with 100% accuracy, its advantage compared to CMR increases as P_isch_ increases. We derived that the difference between the 2 strategies slightly decreases with an increase of the rate of patients with non-diagnostic CMR tests (NDx). In the model, the NDx patients after CMR are oriented to strategy 2 in order to achieve 100% accuracy in these cross-over patients.

**Figure 3:**
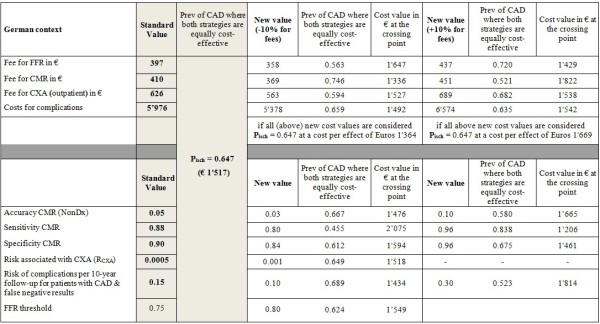
**Costs per patient (Pt) tested in relation to the pre-test likelihood of significant CAD (=P**_**isch**_**) for both strategies.**

**Figure 4:**
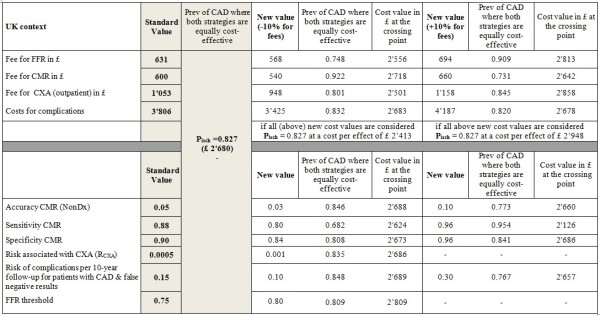
**Results for outpatient procedures performed in the 4 countries.** Costs per effect (= cost-effectiveness) for both strategies in relation to the prevalence of significant CAD (=P_isch_).

Figure 3 shows the effect of pretest likelihood of significant CAD on cost (example for the Swiss health care system). The cost per patient tested increases with increasing pre-test likelihood of significant CAD for both strategies. The costs for CXA + FFR slightly increase as the need for FFR increases with increasing prevalence of significant CAD. For the CMR + CXA strategy the costs increase steeply with increasing pre-test likelihood of significant CAD, since patients positive for ischemia on CMR have to undergo CXA. The two strategies are equally costly for a prevalence of significant CAD of 0.87 (Figure 4). The value for such a crossing point, within the range of P_isch_ (0 – 1.0) depends on the relative costs of the tests and the accuracy of the CMR test (NDx and Sn_CMR_ and Sp_CMR_) (see formulas of costs).

When CXA is considered as inpatient test, the cost per patient tested with strategy 1 (CMR + CXA) is lower than the cost per patient tested with strategy 2 (CXA + FFR) at any level of pre-test-likelihood of CAD.

### Comparison of the cost per effect and of cost-effectiveness for the two strategies

Figure 4 shows the cost per effect, i.e. the cost per patient correctly diagnosed for significant CAD at various levels of CAD prevalence in the 4 countries. We observe that the cost per effect decreases hyperbolically for both strategies as the pretest likelihood increases. The hyperbolic relationship between the prevalence of significant CAD and the costs per patient correctly diagnosed shows the high cost per effect in the patient population with low prevalence of significant CAD (= low P_isch_ values). The costs per effect at low values of P_isch_ are higher for strategy 2 (CX + FFR) than for strategy 1 (CMR + CXA).

By assuming that all tests are outpatient tests, both strategies are equally cost-effective at a pretest likelihood of 62% in Switzerland, 65% in Germany, 83% in the UK, and 82% in the United States with costs of CHF 5,794, € 1,517, £ 2,680, and $ 2,179 per patient correctly diagnosed, respectively. Below this threshold, CMR + CXA shows lower costs per patient correctly diagnosed than CXA + FFR. When the CXA test is performed as an inpatient examination, the crossing point of the two curves shifts towards the right to a prevalence of significant CAD of 77% with costs of CHF 6,819 in Switzerland, to 90% with costs of € 2′847 in Germany, to 93% with costs of £ 4,633 in the UK, and to 94% with costs of $ 3,849 in the US.

### Sensitivity analyses

**Figure 5:**
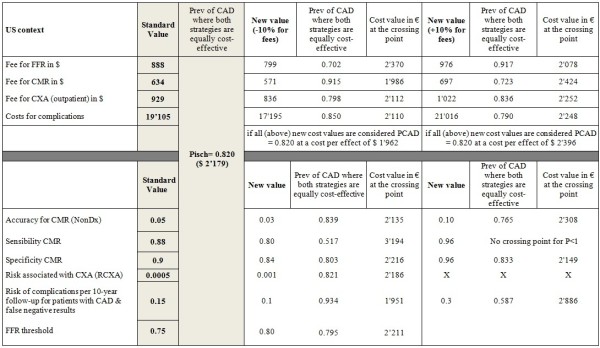
Sensitivity analysis: Switzerland.

**Figure 6:**
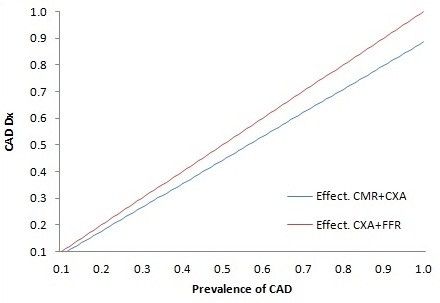
Sensitivity analysis: Germany.

**Figure 7:**
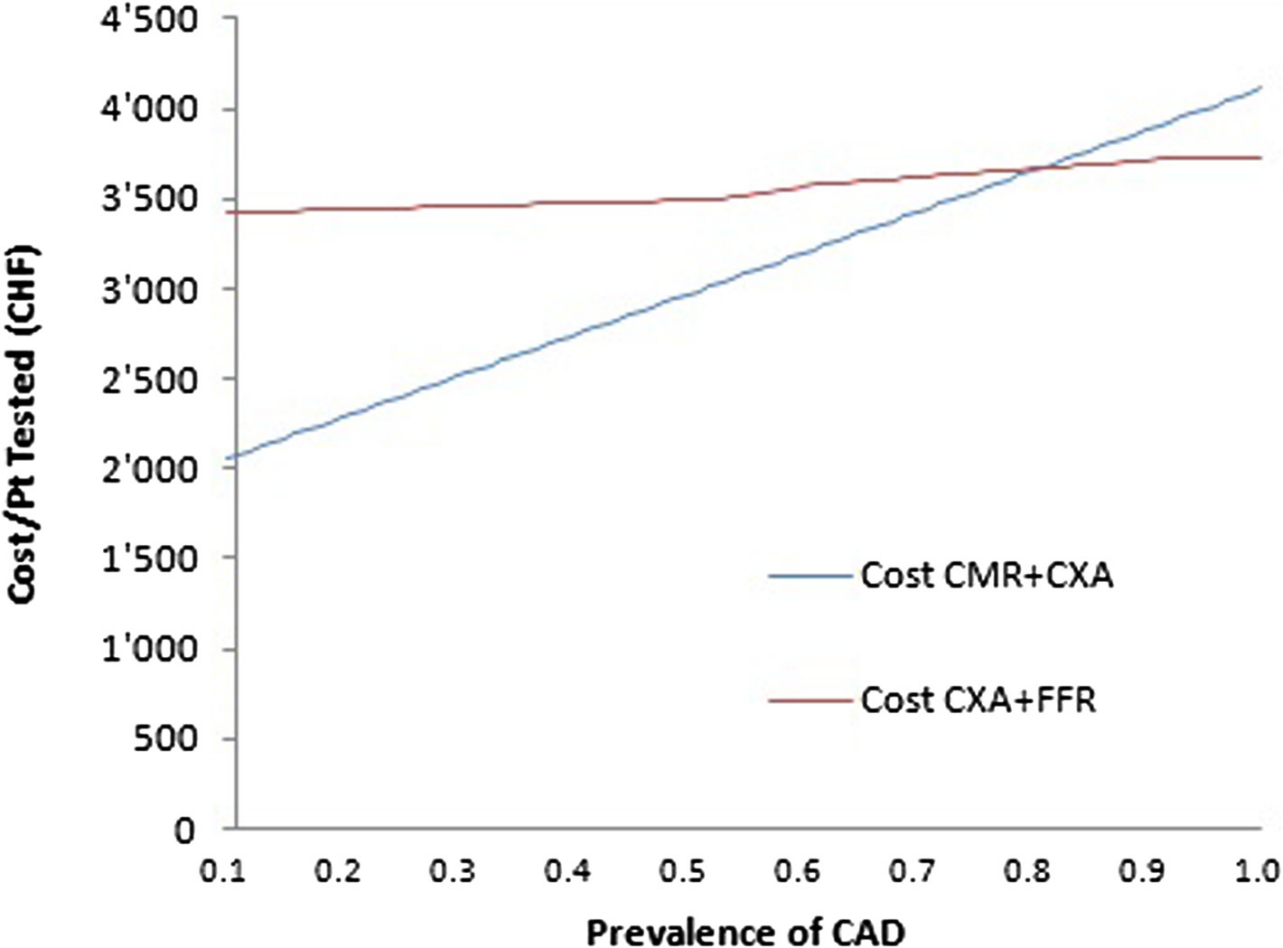
Sensitivity analysis: The United Kingdom.

**Figure 8:**
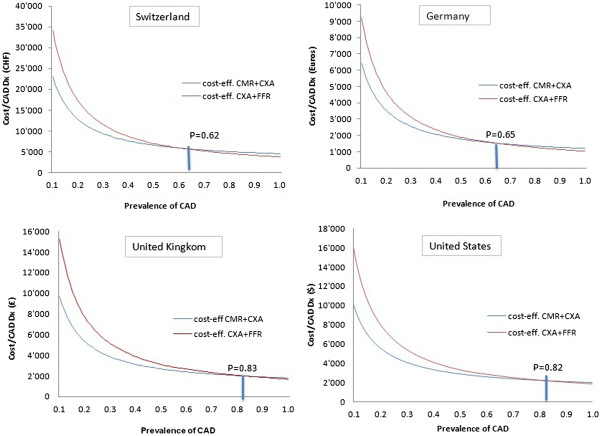
Sensitivity analysis: The United States.

Following a reduction of the sensitivity of the CMR examination by 10% the crossing point shifted to the left by 16, 20, 14, and 30 percentage points for the Swiss, the German, the UK, and the US health care systems, respectively. An increase of the CMR sensitivity by 10% shifted the crossing point to the right by 15, 19, and 12 percentage points for the Swiss, the German, and the UK health care systems, respectively. There is no crossing point for P_isch_ <1 for the US health care system (Figures 5, 6, 7 and 8), i.e. the costs of the CMR strategy are lower than those of the CXA strategy in the US system irrespectively of the pre-test likelihood of CAD.

Changing the specificity of CMR had a minor influence on the crossing point for all health care systems assessed. This was also the case for the other variables tested in the sensitivity analysis except the complications rate. The sensitivity analysis shows that the rate for complications caused by mis-diagnosed CAD, i.e. a lack of detecting CAD, is associated with relevant cost-effectiveness changes at least in the US system. If the rate of complications in false-negative patients by CMR is doubled the crossing point is shifted to the left by 10, 12, 6, and 23 percentage points in the Swiss, German, UK, and US systems, respectively.An increase of the FFR threshold to <0.80 did not substantially influence the cost-effectiveness results as shown in (Figures 5, 6, 7 and 8). The crossing point shifted to the left by 2 or 3 percentage points for the 4 health care systems.

## Discussion

The main findings of this study can be summarized as follows: 1) In all four health care systems analyzed, the cost effectiveness ratio decreases hyperbolically for both diagnostic strategies with an increasing prevalence of hemodynamically significant coronary lesions, i.e. with an increasing prevalence of significant CAD. 2) The increase in the cost-effectiveness for strategy 2, i.e. CXA + FFR, with increasing prevalence of significant CAD is more pronounced than that of the primarily non-invasive CMR + CXA strategy, implying that there is a threshold value of CAD prevalence where strategy 2 becomes more cost-effective than strategy 1. and 3) The crossing point indicating an equal cost-effectiveness for the 2 strategies varied for the 4 countries examined. In Switzerland, strategy 1, i.e. CMR + CXA, is more cost-effective than strategy 2 below a CAD prevalence of 62%. In the German, UK, and US health care systems, a higher cost-effectiveness for CMR + CXA is given for a CAD prevalence below 65%, 83%, and 82%, respectively.

### CAD prevalence for optimum cost-effectiveness of various strategies and current utilization of resources

Current clinical practice guidelines recommend proceeding to PCI only, if relevant myocardial ischemia in symptomatic patients is present [9,10]. Therefore, it appears reasonable to incorporate FFR testing or perfusion CMR for ischemia assessment into models that assess the cost-effectiveness of various strategies suggested for a CAD work-up. Moreover, an FFR-guided PCI approach was shown to be more cost-effective than a simple anatomy-guided, i.e. CXA-based PCI approach [7]. The current results show for all four health care systems assessed, that the pre-test likelihood of CAD is a major factor that influences the cost-effectiveness of a CAD work-up. This is in line with the fact that benefits from diagnostic tests depend on its performance but also on the prevalence of the disease within the evaluated population [35]. According to the current analyses a non-invasive CMR-guided approach is cost-effective for patients with an intermediate pre-test likelihood of disease, which is in line with most guidelines defining intermediate pre-test probabilities as 20-80%. Interestingly, in the US 62% of elective CXA examinations performed in a large sample of approximately 400,000 patients were found to be negative for CAD (stenoses <50% diameter reduction) [18], indicating that in the US the pre-test probability of CAD in daily routine is as low as ~38% which is substantially lower than the calculated threshold of 82%. Similarly, in the UK more than 58% of CXA examinations did not result in PCI or CABG procedures [36] indicating that currently, the pre-test likelihood of significant CAD of patients referred for CXA of 42% is substantially lower than 83% calculated for the UK. In the German health care system, the optimum pre-test likelihood of CAD for a CMR-based strategy is below 65%. However, in 2008, only 43% of patients after CXA were revascularized [17], indicating that an invasive approach was applied in a patient population with a relatively low CAD prevalence of approximately 43%. Finally, in Switzerland, the theoretical threshold for a directly invasive strategy is at 62% CAD prevalence. The portion of normal CXA studies ranged between 55% to 66% over the last 3 years [37], which translates into an approximated pre-test likelihood of significant CAD of 34-45% in Switzerland, thus, again still lower than the prevalence for a calculated optimal cost-effectiveness.

A cost analysis was recently performed based on the data of the German sample of the European CMR registry [38]. Cost savings of 50% were calculated between a CMR strategy and an outpatient invasive CXA strategy which is in line with the fact, that the pre-test probability of CAD in this cohort was 27%, i.e. well below the 65% level. The cost savings for this cohort reported in 2012 would be even higher considering that for these calculations costs for FFR were not yet included. Recently, a cost-effectiveness analysis for UK was performed based on the CE-MARC data [39]. For the prevalence of CAD of 39% in CE-MARC [22], the diagnostic strategy based on CMR (preceded or not by a treadmill test) followed by CXA in case of ischemia on CMR was the most cost-effective strategy of all tested. This finding is well in line with the current calculations which suggest cost-effectiveness for the MR-based approach below a CAD prevalence of 83%. In this context, it should be noted, that in the current study the threshold in favor of a CMR-guided work-up compared cost-effectiveness versus an outpatient CXA + FFR strategy. If inpatient CXA is included into the model, the crossing point shifts towards 77% for Switzerland, and is >90% for Germany, the UK and the US. This indicates that the inpatient CXA + FFR procedures can only compete with non-invasive CMR + CXA for very high rates of CAD prevalence. This result is of even greater importance if we note that in-patient CXA is performed in approximately 67% [40,41], 40% [36], and 88% [42], in the US, the UK, and the German system, respectively.

### Testing for ischemia by invasive *vs* non-invasive techniques

For the current analyses, the FFR technique was assumed to represent the gold standard. The assumption appears justified in the light of a rapidly increasing number of studies confirming the high prognostic value of FFR-guided PCI [5,6,12-14].

At a first glance, a Sn_CMR_ of 88% for ischemia detection (and of Sn_CMR_ = 80% in the sensitivity analysis) may appear relatively high. However, similar and even higher performances were reported with Sn_CMR_/Sp_CMR_ of 82%/94% [43] and Sn_CMR_/Sp_CMR_ of 91%/94% [44]. Importantly, it should be taken into account that these Sn_CMR_ for ischemia detection compare with an ischemia test, i.e. FFR. When lower sensitivities of CMR for ischemia detection are reported, they typically compare perfusion-CMR with coronary anatomy. FFR is generally accepted as a useful tool to guide treatment in CAD patients, as it discriminates patients at risk for complications (FFR-positive) versus those with minimal risk (FFR-negative). In FFR-positive patients, complication rates (death and non-fatal MI) were 3.2%-11.1%/y versus 0.7-7.3%/y in FFR-negative patients [5,6]. In a recent registry-based FFR study, MI in the FFR non-ischemia group was ~1%/y vs ~1.9%/y in the ischemia group [12]. For perfusion CMR, similar discriminative power was observed in approximately 1,700 patients of the EuroCMR registry, with complications rates of 2.7%/y in CMR-positive patients, i.e. in patients with ischemia, versus 0.7%/y in CMR-negative patients [24]. Thus, with this evidence of a similar discriminative power for CMR and FFR, the assumption of FFR being the gold standard, and thus, classifying CMR results differing from FFR as incorrect, might induce a bias towards an underestimation of the cost-effectiveness of the CMR + CXA strategy.

### Limitations

In the four countries, the unit costs for the cardiac examinations fed into the model may vary between different geographical regions and therefore, the results are representative for the entire health care systems under study, but not for smaller geographical regions. In the current model, no treatment costs were included. Correct absence of disease was not directly included in the criterion of effectiveness but the effect was captured indirectly through the costs of complications induced by false negative results. The model does not take into account intangible costs associated with cardiac death. This is because of the third-party-payer-perspective study design. For the US context, we decided to use costs for the material and a reimbursement of the physician to represent FFR costs similar to the approach used by Fearon et al. [7] as the current US reimbursement system does not consider costs for infrastructure nor material. The sensitivity analyses showed a rather moderate effect of prices for FFR on the cost-effectiveness shifting the crossing point by ±6, ±8, ±8, and ±11 percent points for the Swiss, the German, the UK, and the US system, respectively.

Finally, the modeling approach used here implies that some simplifications are built in and the decision process to revascularize or not is reduced to the presence or absence of hemodynamically significant stenoses. It does therefore not consider the clinical background of the patient, which is always important to guide treatment. Accordingly, the presented results might be helpful for trials planning whereas the use of the presented model with real CMR and FFR data acquired in ongoing trials [11] would most likely yield more relevant results.

## Conclusions

With a focus on the latest imaging techniques to detect ischemia, this study shows to what extent the cost-effectiveness of two strategies to diagnose hemodynamically significant coronary lesions, i.e. significant CAD, depends on the prevalence of the disease. The CMR + CXA strategy is more cost-effective than CXA + FFR below a CAD prevalence of 62%, 65%, 83%, and 82% for the Swiss, the German, the UK, and the US health care systems, respectively. These findings may help the decision-making with regard to resource utilization.

## Abbreviations

CAD: Coronary artery disease; CMR: Cardiovascular magnetic resonance; CXA: Invasive x-ray coronary angiography; FFR: Fractional flow reserve; PCI: Percutaneous coronary interventions.

## Competing interests

The authors declare that they have no competing interests.

## Authors' contributions

KM is responsible for the conception and design of the cost-effectiveness analysis; she performed the cost-effectiveness analysis, participated to the data collection and drafted the manuscript. DF participated to data collection and was involved in the calculations of the cost-effectiveness analysis. CP provided advices on the cost-effectiveness analysis. GP provided precisions on how the German health care system works and actively participated in the acquisition of required data in this context. SP provided information on how the UK health care system works and participated in the data acquisition in this context. AW provided explanations on how the US health care system works and participated in the data acquisition in this context. JBW provided explanations on how the Swiss health care system works. JS is responsible for the design of the study, participated to data collection and was involved in the interpretation of the results and drafting the manuscript; he critically revised its intellectual content. In addition, all authors provided helpful comments and relevant suggestions to improve the manuscript and its intellectual content; all authors read and approved the final manuscript.

## Supplementary Material

Additional file 1 **Section A1.** The relationship FFR-Stenosis. **Section A2.** Equations to estimate the costs and the effectiveness for each strategy. Click here for file

Additional file 2 Brief description on how the costs were derived for each country. Click here for file
