# Supplementary material for: Comparative cost-effectiveness analyses of cardiovascular magnetic resonance and coronary angiography combined with fractional flow reserve for the diagnosis of coronary artery disease
Source: J Cardiovasc Magn Reson. 2014 Jan 25;16(1):13. doi: 10.1186/1532-429X-16-13 (PMC4015639; doi:10.1186/1532-429X-16-13)
Supplement: Additional file 1 — Section A1. The relationship FFR-Stenosis. Section A2. Equations to estimate the costs and the effectiveness for each strategy. [file 1532-429X-16-13-S1.doc]

Additional file 1

**Appendix A1. The relationship FFR-Stenosis**

The information used to estimate the relationship between the probability of stenoses ≥50% after a CXA (P_sten_) and the probability of having ischemia after a FFR (P_isch_) < 0.75 was extracted from the 5 articles presented in Table A1 . These articles reported the number of vessels investigated, the tests performed (i.e. CXA and FFR after a positive CXA) and the resulting diagnosis within the investigated vessel (ischemia positive (TP) or negative (FP)) with a threshold to detect ischemia by either an FFR ≤0.75 in a coronary vessel with ≥50% diameter stenosis or the presence of a totally occluded vessel. Assuming that a patient suffers from significant CAD, if at least one of the 3 major coronary vessels presents ischemia, we assessed a discrete relationship between P_isch_ and P_sten_ as shown in Table A1 and Figure A1, which was transferred into a continuous relationship between P_isch_ and P_sten_ by linear interpolation (Figure A1).

Table A1: Relationship between P_isch_ and P_sten_ (vessel and patient levels)

| **Source** | Number of  vessels investigated | True Positive (TP) (FFR<0.75 or vessel totally occluded) | False Positive (FP) | **P_isch_=**  **TP/Nb Vessels** | **P_sten_=**  **TP+FP/Nb Vessels** | **P_isch_**  **Patients** | **P_sten_**  **Patients** |
| --- | --- | --- | --- | --- | --- | --- | --- |
| Meijboom, 2008  JACC | 89 | 11 | 24 | 0.12 | 0.39 | 0.32 | 0.78 |
| Rieber, 2006  European Heart Journal | 129 | 24 | 29 | 0.19 | 0.41 | 0.47 | 0.80 |
| Lockie, 2011  JACC | 126 | 28 | 25 | 0.22 | 0.42 | 0.53 | 0.81 |
| Costa, 2007  JACC | 44 | 12 | 10 | 0.27 | 0.50 | 0.62 | 0.87 |
| Ragosta 2007  American Journal of Cardiology | 88  Total occlusion in 20 vessels | 54 | 34 | 0.61 | 1.00 | 0.94 | 1.00 |


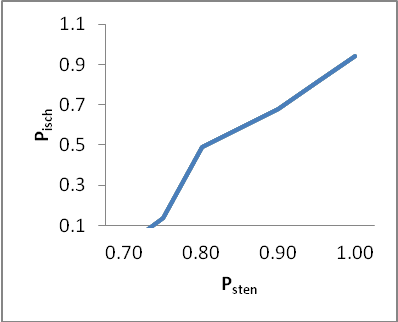
 **Figure A1: Discrete and continuous relationship between** P_isch_ **and** P_sten_ **for a FFR<0.75**

**
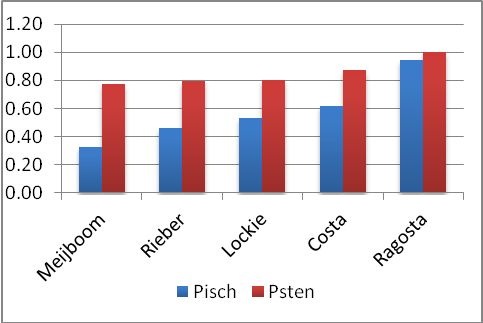
**

Figure A1 shows a positive relationship between the probability for a stenosis ≥50% on CXA (P_sten_) and the probability for ischemia by FFR (P_isch_).

During the last few years the cut-off to define ischemia was raised from an FFR of <0.75 to <0.80. Bettencourt et al. who compared CMR with FFR measurements in 103 patients, found that the prevalence of ischemia increased by ~3% when using an FFR threshold of <0.80 compared to <0.75 . These results reported by Bettencourt et al. did not change significantly the relationship between P_sten_ and P_isch_. In a registry-based study, Li et al. found that 18.1% of all FFR measurements ranged between 0.75 – 0.80 . Assuming that this increased rate is constant over the entire range of CAD prevalence allows us to define the link between P_sten_ and P_isch_ for a threshold of 0.80.

**Appendix A2. Equations to estimate the costs and the effectiveness for each strategy**

**Strategy 1: CMR+CXA**

In strategy 1, a CMR is performed followed by invasive CXA only if CMR is positive


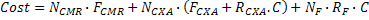
+
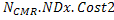


Where

N_CMR_ = number of patients having CMR test

N_CXA_ = number of patients having angiography because of CMR positive

N_F_ = number of patients with false negative CMR

NDx = rate of non diagnosed CMR

P_isch_ : prevalence of coronary artery disease (CAD) in population;

P_sten_ : probability of having a stenosis with Ø >50

F_t_ : fees for the tests

R_F_ rate of complications per 10-year follow-up period for patients with CAD and false-negative tests

C is the complication cost


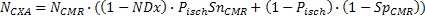


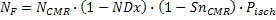


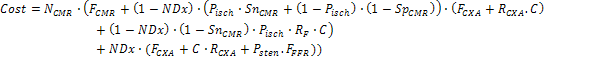


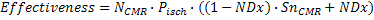


**Strategy 2: CXA+FFR**

In strategy 2, invasive CXA is performed in all patients followed by FFR only if CXA is positive.


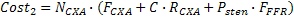


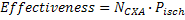


**References**

1. Meijboom WB, Van Mieghem CA, van Pelt N, et al. Comprehensive assessment of coronary artery stenoses: computed tomography coronary angiography versus conventional coronary angiography and correlation with fractional flow reserve in patients with stable angina. J Am Coll Cardiol 2008;52:636-43.

2. Rieber J, Huber A, Erhard I, et al. Cardiac magnetic resonance perfusion imaging for the functional assessment of coronary artery disease: a comparison with coronary angiography and fractional flow reserve. Eur Heart J 2006;27:1465-71.

3. Lockie T, Ishida M, Perera D, et al. High-resolution magnetic resonance myocardial perfusion imaging at 3.0-Tesla to detect hemodynamically significant coronary stenoses as determined by fractional flow reserve. J Am Coll Cardiol 2011;57:70-5.

4. Costa MA, Shoemaker S, Futamatsu H, et al. Quantitative magnetic resonance perfusion imaging detects anatomic and physiologic coronary artery disease as measured by coronary angiography and fractional flow reserve. J Am Coll Cardiol 2007;50:514-22.

5. Ragosta M, Bishop AH, Lipson LC, et al. Comparison between angiography and fractional flow reserve versus single-photon emission computed tomographic myocardial perfusion imaging for determining lesion significance in patients with multivessel coronary disease. Am J Cardiol 2007;99:896-902.

6. Bettencourt N, Chiribiri A, Schuster A, et al. Cardiac magnetic resonance myocardial perfusion imaging for detection of functionally significant obstructive coronary artery disease: A prospective study. International Journal of Cardiology 2013;168:765-773.

7. Li J, Elrashidi MY, Flammer AJ, et al. Long-term outcomes of fractional flow reserve-guided vs. angiography-guided percutaneous coronary intervention in contemporary practice. Eur Heart J 2013.
