# Supplementary material for: Comparative cost-effectiveness analyses of cardiovascular magnetic resonance and coronary angiography combined with fractional flow reserve for the diagnosis of coronary artery disease
Source: J Cardiovasc Magn Reson. 2014 Jan 25;16(1):13. doi: 10.1186/1532-429X-16-13 (PMC4015639; doi:10.1186/1532-429X-16-13)
Supplement: Additional file 2 — Brief description on how the costs were derived for each country. [file 1532-429X-16-13-S2.doc]

Additional file 2

In the following section, a brief description is provided on how the costs of the different tests as well as the costs of the complications were derived for each country. For the estimation of costs of complications several sources of information were used to derive the costs of hospital stays and of rehabilitations. We used reimbursement rates (tariffs) to assess the costs of procedures. Respective figures are given in Table B a-d. Only costs of the public sectors of the four countries were used for model input, costs in the private sector were not analyzed.

**Switzerland**

In Switzerland the outpatient procedures are coded in the TARMED system . The inpatient procedures are coded in the Swiss **D**iagnosis **R**elated **G**roups (DRG) payment system . Costs were derived for this study from the University Hospital of Lausanne (CHUV) for the year 2013.

**Germany**

The outpatient procedures are coded based on a uniform value scale (Einheitlicher Bewertungsmaßstab) based on which costs for this study were calculated. The inpatient procedures are coded in the DRG payment system . The costs of the FFR test were assessed by difference of 2 DRG payments, one including a catheterization + FFR test and one including only the catheterization. The CMR cost was derived from the pre-inpatient situation as published in Moschetti et al. (2012) and was adjusted with an inflation correcting factor to get the 2013 costs .

**The United Kingdom**

With the exception of the CMR, the costs of the different diagnostic procedures were derived from 3 references: 1) the list of procedure codes (OPCS 4.6) ; 2) the national **H**ealth **R**elated **G**roups grouper , and 3) the 2011-2012 reference costs that provide the national average costs for each HRG . The FFR costs were assessed as the difference between 2 HRGs payments: one including the pressure wire procedure and another without it on the basis of an elective inpatient situation. For the CMR costs, the tariff suggested by the British Society of Cardiovascular Magnetic resonance (BSCMR) was applied after adjustment with an inflation correcting factor .

**The United States**

The costs of the tests in the United States were calculated based on the 2013 average national Medicare payments listed by the **C**urrent **P**rocedural **T**erminology codes (CPT) and the assigned **A**mbulatory **P**ayment **C**lassification (APC) category for the outpatient situation and the assigned DRG for the inpatient situation . Since there is no Medicare payment available for the FFR procedure, we simulated it by summing up the costs of the material used and the Medicare average national physician payment assigned to the CPT codes of the test .

**Table B**: Costs of diagnostic tests and complications in the 4 countries

| **Abbreviation** | **Description** |
| --- | --- |
| 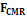 | Costs for CMR |
| 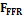 | Costs for FFR |
| 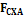 | Costs for CXA |
| 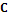_compl_ | Costs of complications (assumed to be a myocardial infarction or stroke with a PCI treatment and a hospital stay of one week) + costs of rehabilitation when covered by the third party payer |

**Table B**a: Costs in Switzerland

| **Abbreviation** | **2013 Payment in CHF** |
| --- | --- |
| 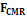 | 1’420 |
| 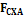 outpatient | 2’508 |
| 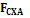inpatient | 4’115 |
| 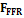 | 1’213 |
| 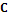_compl_ | 23’087 (15’191+7’896) |

**Table B**b: Costs in Germany

| **Abbreviation** | **2013 Payment in €** |
| --- | --- |
| 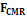 | 410.08 |
| 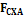 outpatient | 626.1 |
| 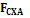inpatient | 2’186 |
| 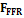 | 397.3 |
| 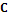_compl_ | 5'976 (3’676+2’300) |

**Table B**c: Costs in the United Kingdom

| **Abbreviation** | **2013 and 2012 Payment in £** |
| --- | --- |
| 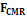 | 600 |
| 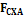 outpatient | 1’053 |
| 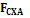inpatient | 2’236 |
| 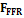 | 631 |
| 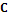_compl_ | 3’806  None costs for the rehabilitation were included since the National Health system does not systematically cover it |

**Table Bd**: Costs in the United States

| **Abbreviation** | **2013 Payment in $** |
| --- | --- |
| 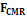 | 633.75 |
| 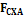 oupatient | 929 |
| 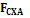inpatient | 2’718 |
| 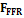 | 887.67 |
| 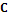_compl_ | 19’105 (11’620+7’485) |

**References**
